# Effectiveness and Costs Associated to Adding Cetuximab or Bevacizumab to Chemotherapy as Initial Treatment in Metastatic Colorectal Cancer: Results from the Observational FABIO Project

**DOI:** 10.3390/cancers12040839

**Published:** 2020-03-31

**Authors:** Matteo Franchi, Donatella Garau, Ursula Kirchmayer, Mirko Di Martino, Marilena Romero, Ilenia De Carlo, Salvatore Scondotto, Giovanni Corrao

**Affiliations:** 1 National Centre for Healthcare Research and Pharmacoepidemiology, 20126 Milan, Italy; 2 Laboratory of Healthcare Research & Pharmacoepidemiology, Department of Statistics and Quantitative Methods, University of Milano-Bicocca, 20126 Milan, Italy; 3 General Directorate for Health, Sardinia Region, 09123 Cagliari, Italy; 4 Department of Epidemiology ASL Roma 1, Lazio Regional Health Service, 00154 Rome, Italy; 5 Department of Medical, Oral and Biotechnological Sciences—Section of Pharmacology and Toxicology, University of Chieti, 66100 Chieti, Italy; 6 Regional Centre of Pharmacovigilance, Regional Health Authority, Marche Region, 60125 Ancona, Italy; 7 Department of Health Services and Epidemiological Observatory, Regional Health Authority, Sicily Region, 90145 Palermo, Italy

**Keywords:** colorectal cancer, target therapy, effectiveness, cost-effectiveness, survival, long-term outcomes

## Abstract

Evidence available on the effectiveness and costs of biological therapies for the initial treatment of metastatic colorectal cancer (mCRC) is scarce and contrasting. We conducted a population-based cohort investigation for assessing overall survival and costs associated with their use in a real-world setting. Healthcare utilization databases were used to select patients newly diagnosed with mCRC between 2010 and 2016. Those initially treated with biological therapy (bevacizumab or cetuximab) added to chemotherapy were propensity-score-matched to those treated with standard chemotherapy alone, and were followed up to June 30th, 2018. Kaplan–Meier survival estimates, restricted mean survival time (RMST) and cumulative costs were compared between the two treatment arms. The study cohort included 1896 mCRC patients treated with biological therapy matched to 5678 patients treated with chemotherapy alone. Median overall survival was 21.8 and 20.2 months, respectively. After 84 months of follow-up, RMSTs were 30.9 and 31.9 months (*p* = 0.193), indicating no differences between the average survival time between treatment arms. Patients treated with biological therapy were associated with higher costs. Cumulative per capita costs were €59,663 and €44,399, respectively. In our study, first-line biological therapy did not improve long-term overall survival and was associated with higher costs as compared to standard chemotherapy.

## 1. Introduction

Bevacizumab (Avastin^®^, Roche, Basel, Switzerland) is a humanized antibody against vascular endothelial growth factor (VEGF). In 2004, the U.S. Food and Drug Administration (FDA) approved its use for the first-line treatment of metastatic colorectal cancer (mCRC) in combination with cytotoxic chemotherapy, one year after the European Medicine Agency (EMA). In 2009, Cetuximab (Erbitux^®^, Merck, Darmstadt, Germany), a chimeric IgG1 monoclonal antibody against epidermal growth factor receptor (EGFR), was made available for the initial treatment of patients with KRAS wild-type mCRC. The efficacy of both these drugs in improving survival and in the prevention of relapses is well documented by randomized controlled clinical trials (RCT) [1,2,3,4,5]. Nevertheless, little and contrasting evidence is available on the benefits of bevacizumab [6,7,8] and cetuximab [9,10] outside RCT settings. In addition, whether the treatment with these therapeutic strategies is cost-effective remains doubtful [11,12,13].

With this premise, a wide population-based cohort investigation was carried out to assess the overall survival (OS) and costs associated with first-line bevacizumab- or cetuximab-based chemotherapy (CT) compared to standard CT alone, in clinical practice. This study is part of an Italian project funded by the Italian Medicines Agency (Agenzia Italiana del Farmaco, AIFA) and the Health Department of the Sardinia Region, which supports the so-called FABIO program (Biologic Drugs in Oncology, the Italian acronym being Farmaci Biologici in Oncologia). The FABIO project is aimed at evaluating the profile of safety, effectiveness, and cost-effectiveness of biologic drugs approved for treating cancer.

## 2. Results

### 2.1. Patients

**Figure 1:**
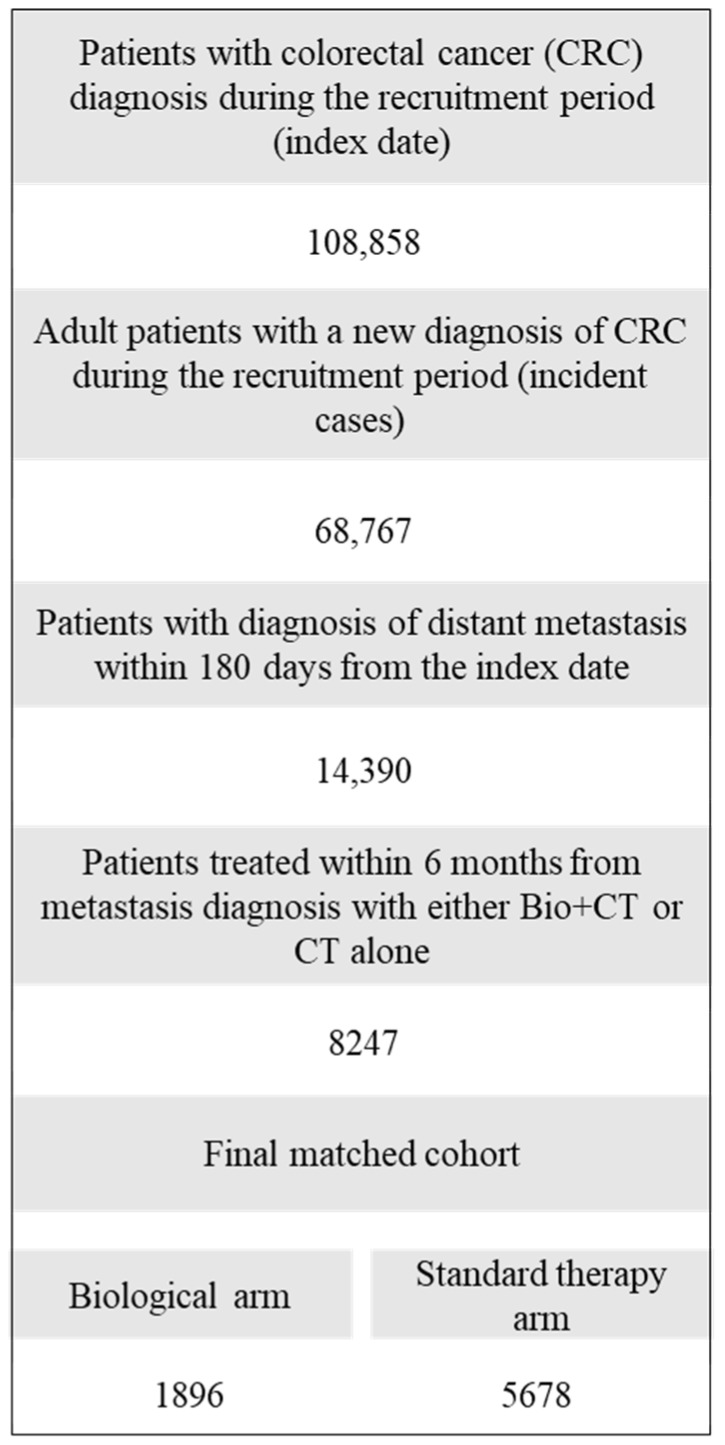
Flowchart of inclusion and exclusion criteria. FABIO project, Italy, 2010–2016.

The process of selection of the entire study cohort is reported in Figure 1 (region-specific data are given in Supplementary Materials, Figure S1). Overall, among 108,858 patients with diagnosis of CRC during the recruitment period, only 8247 met the inclusion criteria, i.e., starting within six months from diagnosis a therapy with biologic (1926; 23.4%) or standard (6321; 76.6%) drugs. Finally, 1896 patients belonging to the biologic arm were matched to 5678 patients on standard CT. Their baseline characteristics are reported in Table 1. Before matching, patients in the biological arm were younger (*p* < 0.001), were more likely to undergo surgery (*p* < 0.001) and had less comorbidities (*p* < 0.001) than patients in the standard arm. After matching, no differences were observed between treatment arms. After a mean follow-up of 27.1 months, 1504 (79.3%) and 4164 (73.3%) deaths occurred in the biologic and standard arms, respectively. 

### 2.2. Effectiveness Profile

**Figure 2:**
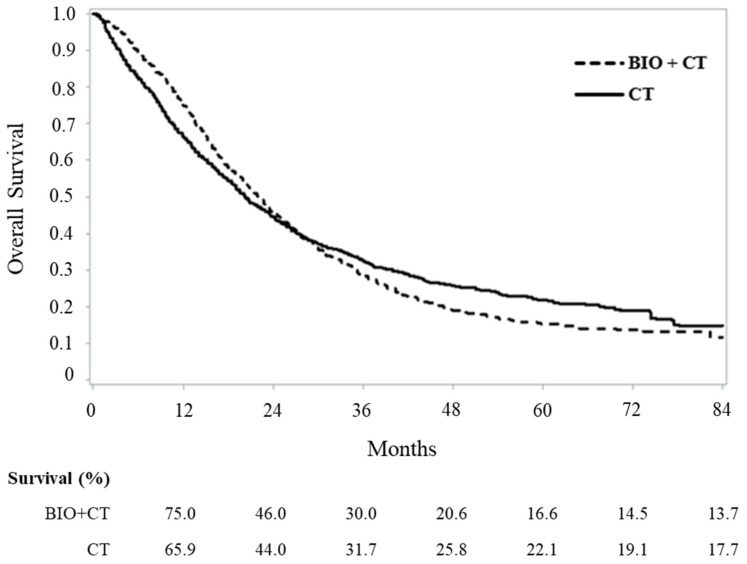
Comparison between Kaplan–Meier overall survival curves of metastatic colorectal cancer cohort members on first-line treatment with biologic-based (bevacizumab or cetuximab, Bio + CT) or standard chemotherapy (CT) alone. FABIO project, Italy, 2010–2016.

Patterns of overall survival experienced by patients on biologic and standard therapy are compared in Figure 2. Survivals at 12, 24, 36 and 84 months from the date of treatment start were 75%, 46%, 30% and 14% among patients on biologic therapy, against 66%, 44%, 32% and 18% among those on standard chemotherapy. Overall, these data correspond to median survivals of 21.8 and 20.2 months, and RMST values of 30.9 (95% CI: 29.6 to 32.1) and 31.9 (31.0 to 32.6) months (*p* = 0.193). RMST figures during the first two years after starting treatment were 17.8 and 16.5 months, respectively (*p* < 0.001). Region-specific survival curves substantially confirmed the national data (please see Supplementary Materials, Figure S2, Table S1).

### 2.3. Comparing Healthcare Costs

**Figure 3:**
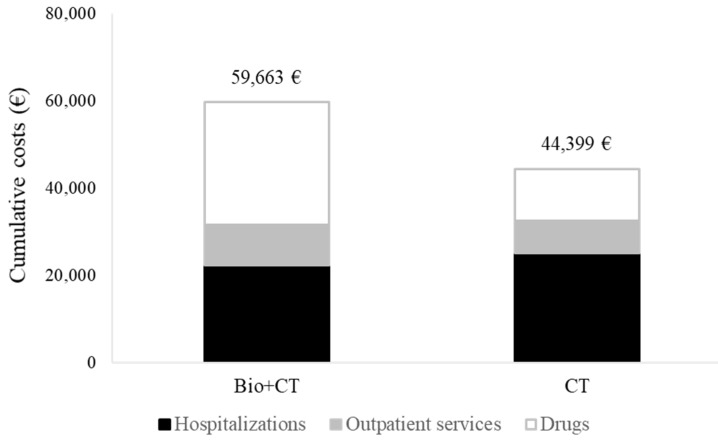
Comparison between cumulative per capita healthcare costs sustained by the NHS for caring for metastatic colorectal cancer cohort members on first-line treatment with biologic-based (bevacizumab or cetuximab, Bio + CT) or standard chemotherapy (CT) alone. FABIO project, Italy, 2010–2016.

**Figure 4:**
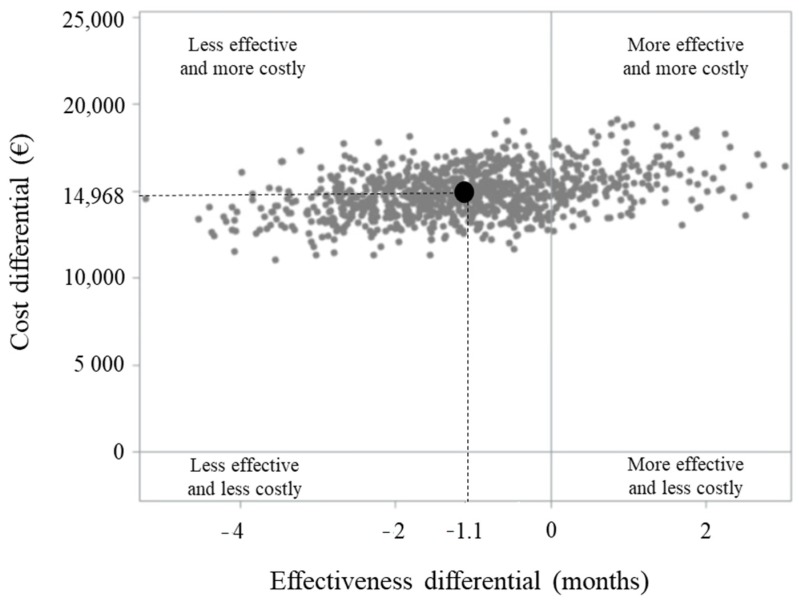
Incremental cost-effectiveness ratio (ICER) scatterplot comparing metastatic colorectal cancer cohort members on first-line treatment with biologic-based (bevacizumab or cetuximab, Bio + CT) or standard chemotherapy (CT) alone. FABIO project, Lombardy Region, 2010–2014.

Cumulative NHS healthcare costs according to therapeutic strategy are shown in Figure 3. On average, €59,663 and €44,399 were spent for each patient belonging to the biologic and standard arms, respectively, within the first 60 months after starting therapy. The average cost of a patient on treatment with biologic therapy included €22,287 for hospitalization, €9269 for outpatient services and €28,107 for drugs. Corresponding figures for a patients on treatment with standard chemotherapy were €25,193, €7375 and €11,831, respectively. The cost-effectiveness profiles shown in Figure 4 confirm that looking at a long-term time horizon of 5 years, initiating therapy with a biologic drug was neither effective (as the patients on the biologic arm experienced a reduced survival of 1.1 months) nor cost-effective (involving biologic therapy had an additional cost of €14,506), generating a negative incremental cost-effectiveness ratio (ICER) of €13,187. Less effective and more costly profiles occurred in 81% of the 1000 bootstrap replications. A cost-effectiveness profile measured during the first two years after starting treatment is shown in Supplementary Materials, Figure S3.

## 3. Discussion

Our study showed that among 8247 patients with colorectal cancer already metastatic at diagnosis who started drug therapy within six months after diagnosis, not even one out of four (23.4%) received biologic therapy with bevacizumab or cetuximab, while the remaining 76.6% were treated with standard chemotherapy. In addition, our study confirmed the findings of several RCT, offering evidence that biologic therapy leads to short-term benefits (i.e., during the first two years) [1,2,3,4,5]. The new important finding, however, is that in clinical practice, biologic therapy did not lead to any long-term benefit in terms of average survival time, and involved a considerable amount of additional healthcare costs. These results provide the largest available piece of evidence on long-term effectiveness and cost-effectiveness profiles of first-line biologic therapy in mCRC patients in a real-life setting.

As far as the effectiveness profile is concerned, a recent observational study conducted in some districts of Lombardy and Sicily Italian regions showed no significant reduction in all-cause mortality (−14%; 95% CI from −44% to +33%) in propensity score (PS)-matched mCRC patients treated with first-line bevacizumab-based therapy, as compared to chemotherapy alone, after a 3-year follow-up [6]. An observational study of U.S. mCRC patients showed that, while a 5-year beneficial effect of first-line bevacizumab was observed when added to FOLFOX/FOLFIRI/IFL (*p* = 0.003) or to irinotecan only (*p* = 0.03), this effect disappeared after about three years from treatment start [7].

As far as healthcare costs are concerned, a recent Italian study showed mCRC patients treated with bevacizumab or cetuximab combined with standard CT (i.e., capecitabine, FOLFOXIRI, XELOX or FOLFOX4) had direct costs ranging from 16 thousand euros to 43 thousand euros, assuming a duration of 6.1 months for each treatment regimen [14]. These data are not so far from our cost estimate which is around 60 thousand euros in a 60-month timespan. Moreover, our analysis showed that costs due to hospitalizations and outpatient services were similar between treatment arms. However, drug costs were markedly higher in the biological arm, as compared to the standard chemotherapy arm. 

Finally, as far as the cost-effectiveness profile is concerned, our findings are consistent with recent studies showing that (i) adding bevacizumab to first-line chemotherapy in mCRC patients was not cost-effective in five countries, including the U.S., the U.K., Canada, Australia and Israel [11]; (ii) the cost-effectiveness value of cetuximab in K-RAS wild-type previously untreated mCRC patients is poor in the U.K. [13].

Our study has several strengths. First, to the best of our knowledge, this is the largest study performed so far comparing survival of mCRC patients treated first-line with biological therapy or traditional therapy. Second, the extended length of follow-up allowed us to evaluate long-term effectiveness and costs associated with biological therapy. Third, the target population from which we selected the final cohort was representative of the routine clinical practice in Italy. Indeed, all the beneficiaries of the NHS hospitalized with a diagnostic code of CRC and a concurrent (i.e., during the following six months) code of distant metastasis during the recruitment period were included in the study, with no restrictions on age and concomitant diseases. Moreover, participant regions covered approximately 42% of the Italian population and likely reflected the heterogeneity in clinical practice for treating mCRC. When comparing region-specific survival curves, some differences appear between regions. This may reflect the heterogeneity in the management of colorectal cancer in different geographical areas. Indeed, despite the Italian NHS providing universal coverage for many aspects of healthcare, including those for colorectal cancer, regional disparities likely reflect differences in the quality of care provided by public services. All regions participating in the study were requested to provide the availability of a minimum set of information and covariates necessary for the harmonization of both the study design and the statistical analysis, thus minimizing the potential bias due to the use of different criteria between regions. However, we cannot exclude that the observed differences may be explained, at least in part, by the different quality of data among regions.

On the other hand, the main limitation of the study is the paucity of data on individual characteristics, clinical features and drug patterns and regimens. Indeed, since patients were not randomly allocated to first-line biological therapy or standard therapy, the results may be affected by confounding. That is, patterns of OS observed in this study might have been generated by factors influencing both therapeutic strategy and the baseline risk of death. Factors such as ethnicity or socioeconomic status can be confidently ruled out because the Italian population is largely Caucasian and free-of-pay access to cancer care is ensured for all NHS beneficiaries. In addition, with the aim of better taking into account measurable confounders, a propensity score matching design was used. Other unmeasured factors, however, might affect our conclusions. For example, information on clinical features (e.g., ECOG performance status, KRAS/BRAF mutation, primary tumor location) and therapeutic regimens (e.g., FOLFIRI, FOLFOX) given in combination with biological therapy were not available in administrative databases.

## 4. Materials and Methods 

### 4.1. Data Sources

The Italian National Health Service (NHS) provides universal and mostly free-of-charge healthcare services, including medicines for cancer. The service is administered within each of the 21 Italian regions by an automated system of healthcare utilization (HCU) databases that collect a variety of information, at least including: (i) demographic and administrative data of NHS beneficiaries (practically the entire resident population); (ii) hospital discharge records providing information on primary diagnosis, co-existing conditions and procedures performed to inpatients admitted in public and private hospitals and coded according to the International Classification of Diseases, 9th Revision Clinical Modification (ICD-9-CM) classification system; (iii) drugs dispensed by territorial pharmacies and medicines directly administered in the outpatient setting and day-hospitals, coded according to the Anatomical Therapeutic Chemical (ATC) classification system; (iv) data on outpatient services, including specialist visits, laboratory tests and diagnostic imaging, and (v) co-payment exemption database, including exemption for cancer, the latter two both coded according to the national nomenclature. Record-linkage between databases is allowed through a single identification code (Regional Health Code). In order to preserve privacy, each identification code is automatically converted into an anonymous code, and the inverse process is prevented by deletion of the conversion table.

### 4.2. The FABIO Network

The FABIO program was conducted by retrieving HCU data from six Italian regions localized in Northern (Lombardy), Central (Lazio and Marche), Southern (Abruzzo) and Insular (Sardinia and Sicily) Italy. The corresponding resident population overall accounts for about 25.5 million inhabitants, representing 42.1% of the entire Italian population.

### 4.3. Designing, Harmonizing and Achieving Regional Cancer Research Platforms

A regional cancer research platform (RCRP) within each region participating in the FABIO network was built according to the following procedure. First, although HCU databases did not substantially differ, a between-region data harmonization was performed, thus allowing data extraction processes to be targeted at the same semantic concepts. Second, NHS beneficiaries who leave their ‘footprints’ suggestive of cancer through specific services (e.g., at least an ICD9-CM diagnostic code of cancer was issued, and/or an ATC code of cancer drug was administered, and/or the co-payment exemption due to cancer was left) were identified. Third, the identification code of NHS beneficiaries identified in the previous phase served for catching all the services provided to patients likely affected by cancer. In summary, each of the six RCRPs contain interconnectable HCU data of NHS beneficiaries likely affected by cancer in a given time span. Depending on data availability, RCRP differences may eventually regard the time-window depth covered by administrative recording. Fourth, RCRP data may be extracted and processed according to standardized protocols developed after discussion and consensus of the study Steering Committee. Finally, protocols are translated into a common Statistical Analysis System (SAS) or R script written by the biostatistician responsible of the FABIO program (MF). In this way, the program required data to be handled and analyzed within each region, and summarized regional-level aggregate results, so to guarantee data protection and to comply with regional rules.

### 4.4. Cohort Selection, Exposure Definition and Follow-Up

Specific diagnostic and therapeutic codes used for the current study are given in Supplementary Materials (Table S2).

During the recruitment period, which varied in the timespan between 2010 and 2016, based on data availability of participating regions, NHS beneficiaries with a diagnosis of mCRC were selected. The date of the first hospital admission for colorectal cancer was defined as the “index date”. In order to select only incidental cases, patients were excluded if they received diagnosis of malignancy and/or underwent chemotherapy within five years before the index date. Patients were also excluded if, at the index date, they were younger than 18 years or died during the index hospitalization. Among the remaining patients, those who had a diagnosis of distant metastasis and who received drug therapy with either biologic drugs (i.e., bevacizumab or cetuximab) or standard CT within six months after the index date were included in the study cohort. The date of the first cancer drug dispensation after mCRC diagnosis was defined as “treatment start”. Cohort members were classified as exposed to first-line biologic therapy (i.e., belonging to the biologic therapy arm), or to standard CT alone (i.e., belonging to the standard arm), according to whether during 21 days following the treatment start they did or did not receive at least a biologic dispensation, respectively. A recent published observational study conducted on mCRC patients in Italy showed that 21 days (i.e., the duration of a chemotherapy cycle) are sufficient for identifying patients who start biological therapy, excluding those who received chemotherapy alone [6]. Each cohort member accumulated person-years of follow-up from the date of treatment start until death (i.e., the outcome of interest), or censoring (emigration, or end-point of follow-up), whichever came first. End-point of follow-up was the last date with data available within each region (i.e., in a timespan between December 31, 2016 and June 30, 2018).

### 4.5. Covariates

Baseline covariates included age, sex, year of mCRC diagnosis and surgery in the timespan between the dates of colorectal cancer diagnosis and treatment start. In addition, the so-called Multisource Comorbidity Score (MCS), a simple score recently developed and validated in Italy [15], was used for assessing the general clinical profile of each cohort member. In the current study, the weights of the conditions that contribute to the score were recalculated by considering the cohort of cancer patients, rather than the general population as in the original version of the MCS [16].

### 4.6. Matching Cohort Arms

In order to reduce the between-treatments heterogeneity, a propensity score (PS)-matched analysis was performed [17]. A logistic regression for the association between exposure (to the covariates listed above and the 25 conditions contributing to MCS) and response (i.e., the therapeutic strategy with biologic or standard drugs) was used for PS estimates. Each patient belonging to the biologic arm (index case) was matched with up to four patients randomly selected from those on the standard arm with the same PS value of the corresponding index case, with a difference of ±0.01 tolerated.

### 4.7. Statistical Analyses

Between-arm differences in baseline characteristics were tested by the chi-square statistics using its version for trend or the Fisher exact test where appropriate. Overall survival was estimated by using the Kaplan–Meier (KM) estimator. In order to increase the precision of the estimates, between-region summarized KM curves were estimated. As regional data were not available to be analyzed in a pooled analysis, a method for reconstructing individual patient data starting from each regional KM curve was applied. Briefly, a digital software was used to read the coordinates of KM curves within each region. Information on the number of patients still alive at each year of follow-up and the total number of deaths was used to solve the inverted KM equation, which allowed for the reconstruction of regional data for each arm, to obtain pooled individual patient data [18]. Median survivals were reported as descriptive measures of survival in the two treatment arms. However, median survival does not capture the long-term survival profile well, being insensitive to long-term survivors. Thus, the estimate of the difference in median survivals can result in an inconsistent conclusion about the treatment effect [19]. Moreover, the standard measures typically used for comparing the risk of death among treatment arms (i.e., the log-rank test and the hazard ratio) are only valid when the proportional hazard assumption is verified. Since, in our cohort, the Kaplan–Meier curves of the two treatment arms crossed, indicating that the proportional assumption was violated, the restricted mean survival time (RMST) was used for interpreting treatment effect. RMST, that is the area under the Kaplan–Meier curve, represents the average survival time (expressed in months) experienced by cohort members [19,20,21]. 

Finally, cumulative healthcare costs (CHC) according to the drug therapy strategy were calculated by means of the Bang and Tsiatis estimator [22], a method that takes into account censored cost data. For each patient, CHC was calculated by summing up direct costs incurred by the NHS regional authority for inpatient and outpatient services and drug dispensations supplied during follow-up. Between-region summarized cumulative costs were estimated by weighting within-region costs by the number of patients still alive at the end of each month of follow-up. The Lazio region did not contribute to the costs analysis, since no information on drug costs was available at the time of the study analyses. 

Finally, limited to data from the Lombardy region (i.e., the largest region among those included in the FABIO program), the incremental cost-effectiveness ratio (ICER) was measured by dividing the differences in healthcare costs (CHC) and health-related outcomes (RMST) between the two treatment arms (biological arm and standard arm). The ICER is the healthcare expenditure expected to be saved (or added, depending on the sign) for gaining one month of life due to starting therapy with a biological drug. A non-parametric bootstrap method based on 1000 re-samples [23] was used to explore the uncertainty in the cost-effectiveness estimates [24].

For all the tested hypotheses, two-tailed *p*-values less than 0.05 were considered statistically significant.

### 4.8. Ethical Issues

The Ethical Committee of the University of Milano-Bicocca approved the protocol No. 506, entitled "Valutazione dell’utilizzo dei farmaci biologici nel paziente oncologico: progetto FABIO (Farmaci Biologici in Oncologia)” and established that the study (i) was exempt from informed consent (according to General Authorization for the Processing of Personal Data for Scientific Research Purposes Issued by the Italian Privacy Authority on December 15, 2016; http://www.garanteprivacy.it/web/guest/home/docweb/-/docweb-display/docweb/5805552), (ii) provided sufficient guarantees of individual records anonymity, and (iii) was designed according to quality standards of good practice of observational research based on secondary data.

## 5. Conclusions

In summary, first-line therapy with bevacizumab or cetuximab in combination with standard chemotherapy for patients with colorectal cancer already metastatic at diagnosis is associated with modest short-term survival gaining (which, however, is annulled after two years from starting therapy) and entails high healthcare costs. On balance, our study suggests that biological therapy has marginal added value in the first-line treatment of patients with metastatic colorectal cancer.

## Figures and Tables

**Table 1:** Comparison between baseline characteristics of metastatic colorectal cancer patients belonging to the original and propensity score (PS)-matched cohorts on first-line treatment with biologic-based (bevacizumab or cetuximab) or standard chemotherapy alone. FABIO project, Italy, 2010–2016.

Characteristic	Original Cohort Members		PS-Matched Cohort Members	
	Bevacizumab- or cetuximab-based therapy (N = 1926)	Standard chemotherapy only (N = 6321)	Bevacizumab- or cetuximab-based therapy (N = 1896)	Standard chemotherapy only (N = 5678)
Sex				
Men	1109 (57.6)	3608 (57.1)	1107 (58.4)	3316 (60.4)
Women	817 (42.4)	2713 (42.9)	789 (41.6)	2362 (39.6)
*p*-value^†^	0.697		0.991	
Age at diagnosis (years)				
18–49	238 (12.4)	505 (8.0)	237 (12.5)	713 (12.6)
50–59	441 (22.9)	1049 (16.6)	425 (22.4)	1386 (24.4)
60–69	669 (34.7)	1826 (28.9)	648 (34.2)	1866 (32.9)
≥70	578 (30.0)	2941 (46.5)	586 (30.9)	1713 (30.2)
*p*-value^†^	<0.001		0.339	
Surgery at index hospital admission				
Yes	1303 (67.7)	3614 (57.2)	1300 (68.6)	3815 (67.2)
No	623 (32.3)	2707 (42.8)	596 (31.4)	1863 (32.8)
*p*-value ^†^	<0.001		0.268	
Comorbidities (MCS) ^‡^				
0–2	1110 (57.6)	2839 (44.9)	1110 (58.6)	3304 (58.2)
3–5	632 (32.8)	2077 (32.9)	627 (33.1)	1913 (33.7)
6–8	147 (7.6)	757 (12.0)	126 (6.6)	322 (5.7)
≥9	38 (2.0)	649 (10.3)	33 (1.7)	139 (2.4)
*p*-value ^†^	<0.001		0.132	

^†^ Chi-square test, or its version for the trend, or Fisher exact test, where appropriate; ^‡^ MCS: Multisource Comorbidity Score.

## Supplementary Materials

The following are available online at https://www.mdpi.com/2072-6694/12/4/839/s1, Figure S1: Flow-chart of inclusion and exclusion criteria according to the Italian region where patients were diagnosed. FABIO project, Italy, 2010–2016; Figure S2: Kaplan–Meier overall survival curves of metastatic colorectal cancer cohort members on first-line treatment with biologic-based (bevacizumab or cetuximab, dotted line) or standard chemotherapy (continuous line) alone in Lombardy (**A**), Lazio (**B**), Marche (**C**), Abruzzo (**D**), Sardinia (**E**) and Sicily (**F**) regions. FABIO project, Italy, 2010–2016; Figure S3: ICER scatterplot measured during the first two years after starting treatment comparing metastatic colorectal cancer cohort members on first-line treatment with biologic-based (bevacizumab or cetuximab, Bio + CT) or standard chemotherapy (CT) alone. FABIO project, Lombardy region, 2010–2014; Table S1: Region-specific restricted mean survival time (RMST) and median overall survival (OS) of metastatic colorectal cancer cohort members on first-line treatment with biologic-based (bevacizumab or cetuximab; Bio + CT) or standard chemotherapy (CT) alone. FABIO project, Italy, 2010–2016, Table S2: ICD-9 CM and ATC codes of diseases/conditions and drugs used for the current study.
