# Supplementary material for: Effectiveness and Costs Associated to Adding Cetuximab or Bevacizumab to Chemotherapy as Initial Treatment in Metastatic Colorectal Cancer: Results from the Observational FABIO Project
Source: Cancers (Basel). 2020 Mar 31;12(4):839. doi: 10.3390/cancers12040839 (PMC7226266; doi:10.3390/cancers12040839)
Supplement: Supplementary file 1 [file cancers-12-00839-s001.pdf]

Supplementary Materials

Table S1. Region-specific restricted mean survival time (RMST) and median overall survival (OS) of metastatic colorectal cancer cohort members on first-line treatment with biologic-based (bevacizumab or cetuximab; Bio + CT) or standard chemotherapy (CT) alone. FABIO project, Italy, 2010–2016.

Region	RMST (Months)			Median OS (Months)	
	**Bio + CT**	**CT**	*p*-value	Bio + CT	CT
Lombardy	31.8	32.9	0.392	20.5	16.8
Lazio	31.1	31.2	0.956	25.4	23.0
Marche	16.9	20.9	0.479	10.9	17.6
Abruzzo	26.7	31.1	0.319	18.8	25.4
Sardinia	20.8	20.0	0.622	21.0	22.7
Sicily	26.8	26.1	0.549	20.5	18.9

Table S2. ICD-9 CM and ATC codes of diseases/conditions and medicaments drugs used for the current study.

**Disease/Condition**	**ICD9-CM Codes**
Colorectal cancer	153, 154.0, 154.1, 154.8
Any malignant cancer	140–208
Chemotherapy	V58.1, 99.25, 99.28, 00.10
Distant metastasis	197, 198, 199
Colorectal surgery	32.3, 34.59, 42.86, 44.39, 45.41, 45.43, 45.49, 45.51, 45.61, 45.62, 45.71–45.76, 45.79, 45.8, 45.90–45.94, 46.01–46.04, 46.10, 46.11, 46.13, 46.14, 46.20–46.23, 46.40, 46.43, 46.51, 46.52, 46.76, 46.79, 46.85, 46.93, 46.94, 48.33, 48.35, 48.49, 48.5, 48.62, 48.63, 48.69, 48.79, 50.22, 50.29, 50.3, 54.12, 54.4, 54.51, 54.59, 54.61, 68.8
**Drug**	**ATC codes**
Bevacizumab	L01XC07
Cetuximab	L01XC06
Any oncologic drug	L01

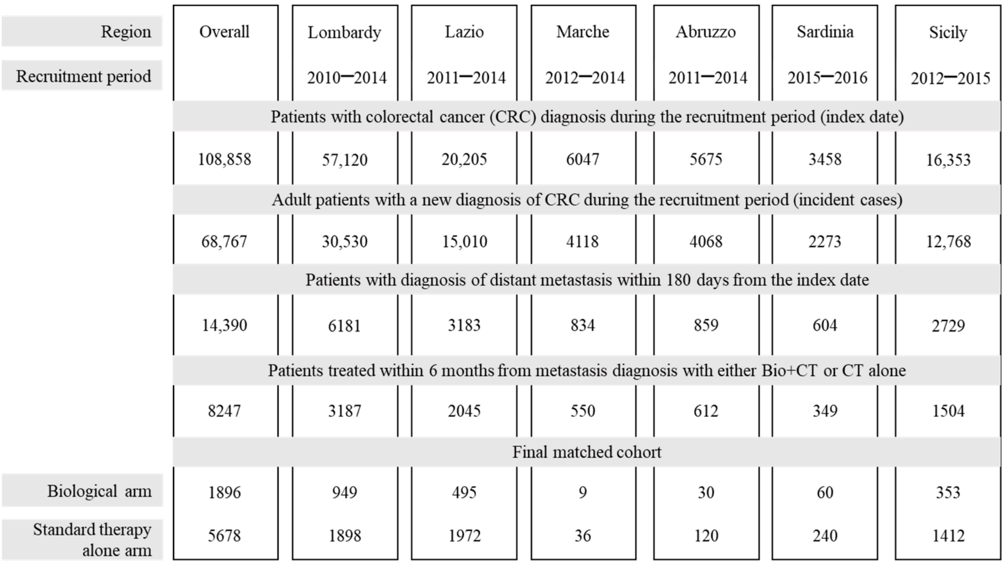

Figure S1. Flow-chart of inclusion and exclusion criteria according with the Italian region where patients were diagnosed. FABIO project, Italy, 2010–2016.

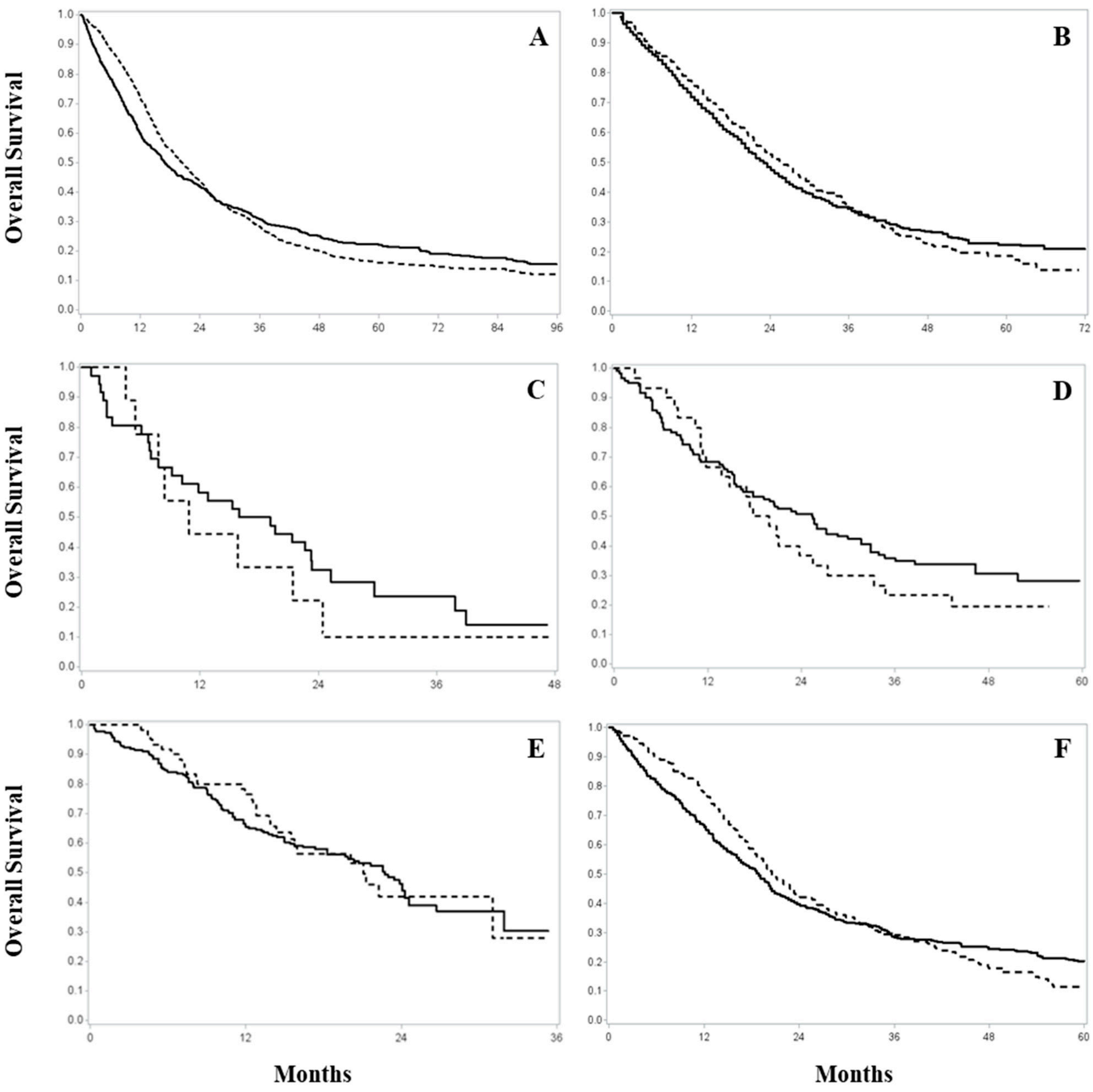

Figure S2. Kaplan-Meier overall survival curves of metastatic colorectal cancer cohort members on first-line treatment with biologic-based (bevacizumab or cetuximab, dotted line) or standard chemotherapy (continuous line) alone in Lombardy (**A**), Lazio (**B**), Marche (**C**), Abruzzo (**D**), Sardinia (**E**) and Sicily (**F**) Region. FABIO project, Italy, 2010–2016.

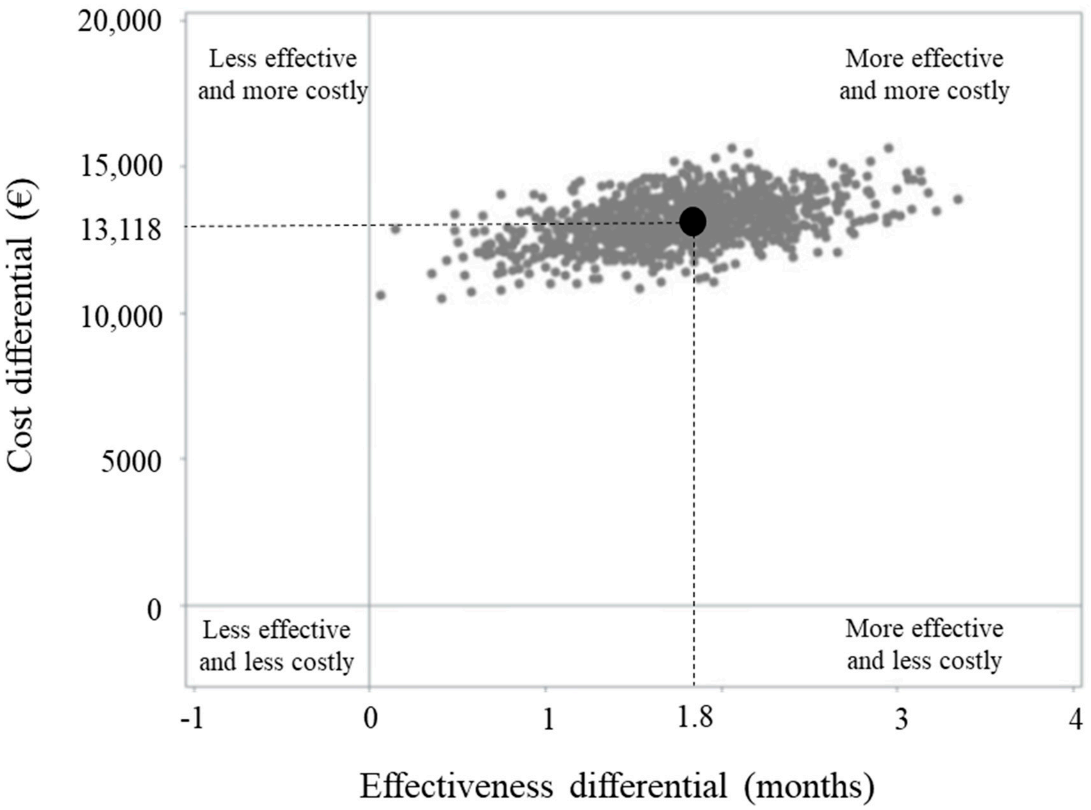

Figure S3. ICER scatterplot measured during the first two years after starting treatment comparing metastatic colorectal cancer cohort members on first-line treatment with biologic-based (bevacizumab or cetuximab, Bio+CT) or standard chemotherapy (CT) alone. FABIO project, Lombardy Region, 2010–2014.
